# Research progress on the PEGylation of therapeutic proteins and peptides (TPPs)

**DOI:** 10.3389/fphar.2024.1353626

**Published:** 2024-03-08

**Authors:** Chunxiao Li, Ting Li, Xinya Tian, Wei An, Zhenlong Wang, Bing Han, Hui Tao, Jinquan Wang, Xiumin Wang

**Affiliations:** ^1^ Institute of Feed Research, Chinese Academy of Agricultural Sciences, Beijing, China; ^2^ Key Laboratory of Feed Biotechnology, Ministry of Agriculture and Rural Affairs, Beijing, China; ^3^ State Key Laboratory of Pathogen and Biosecurity, Beijing Institute of Biotechnology, Beijing, China

**Keywords:** PEGylation, therapeutic protein, antimicrobial peptides, antibodies, modification

## Abstract

With the rapid advancement of genetic and protein engineering, proteins and peptides have emerged as promising drug molecules for therapeutic applications. Consequently, there has been a growing interest in the field of chemical modification technology to address challenges associated with their clinical use, including rapid clearance from circulation, immunogenicity, physical and chemical instabilities (such as aggregation, adsorption, deamination, clipping, oxidation, etc.), and enzymatic degradation. Polyethylene glycol (PEG) modification offers an effective solution to these issues due to its favorable properties. This review presents recent progress in the development and application of PEGylated therapeutic proteins and peptides (TPPs). For this purpose, firstly, the physical and chemical properties as well as classification of PEG and its derivatives are described. Subsequently, a detailed summary is provided on the main sites of PEGylated TPPs and the factors that influence their PEGylation. Furthermore, notable instances of PEG-modified TPPs (including antimicrobial peptides (AMPs), interferon, asparaginase and antibodies) are highlighted. Finally, we propose the chemical modification of TPPs with PEG, followed by an analysis of the current development status and future prospects of PEGylated TPPs. This work provides a comprehensive literature review in this promising field while facilitating researchers in utilizing PEG polymers to modify TPPs for disease treatment.

## 1 Introduction

With the development of gene recombinant technology, therapeutic proteins and peptides (TPPs) currently account for 10% of the global pharmaceutical market and are projected to exceed US$70 billion annually due to their distinctive attributes, including high specificity, potent bioactivity, and minimal side effects (Wang et al., 2002; Leader et al., 2008; Manning et al., 2010; Du et al., 2015). The Food and Drug Administration (FDA) has granted clinical approval for more than 239 medicinal TPPs. However, despite their increasing utilization in clinical practice, most TPPs suffer from several drawbacks such as low solubility, poor stability, short half-life, and high immunogenicity that compromise their efficacy and restrict their therapeutic applications (Veronese and Pasut, 2005). Chemical modification emerges as a robust strategy to enhance the stability, solubility, and reduce the immunogenicity profile of TPPs (Braman, 2002; Sung et al., 2003; Qin et al., 2005; Veronese and Mero, 2008). Notably, significant attention has been directed towards developing novel polymers aimed at improving the properties of TPPs.

**FIGURE 1:**
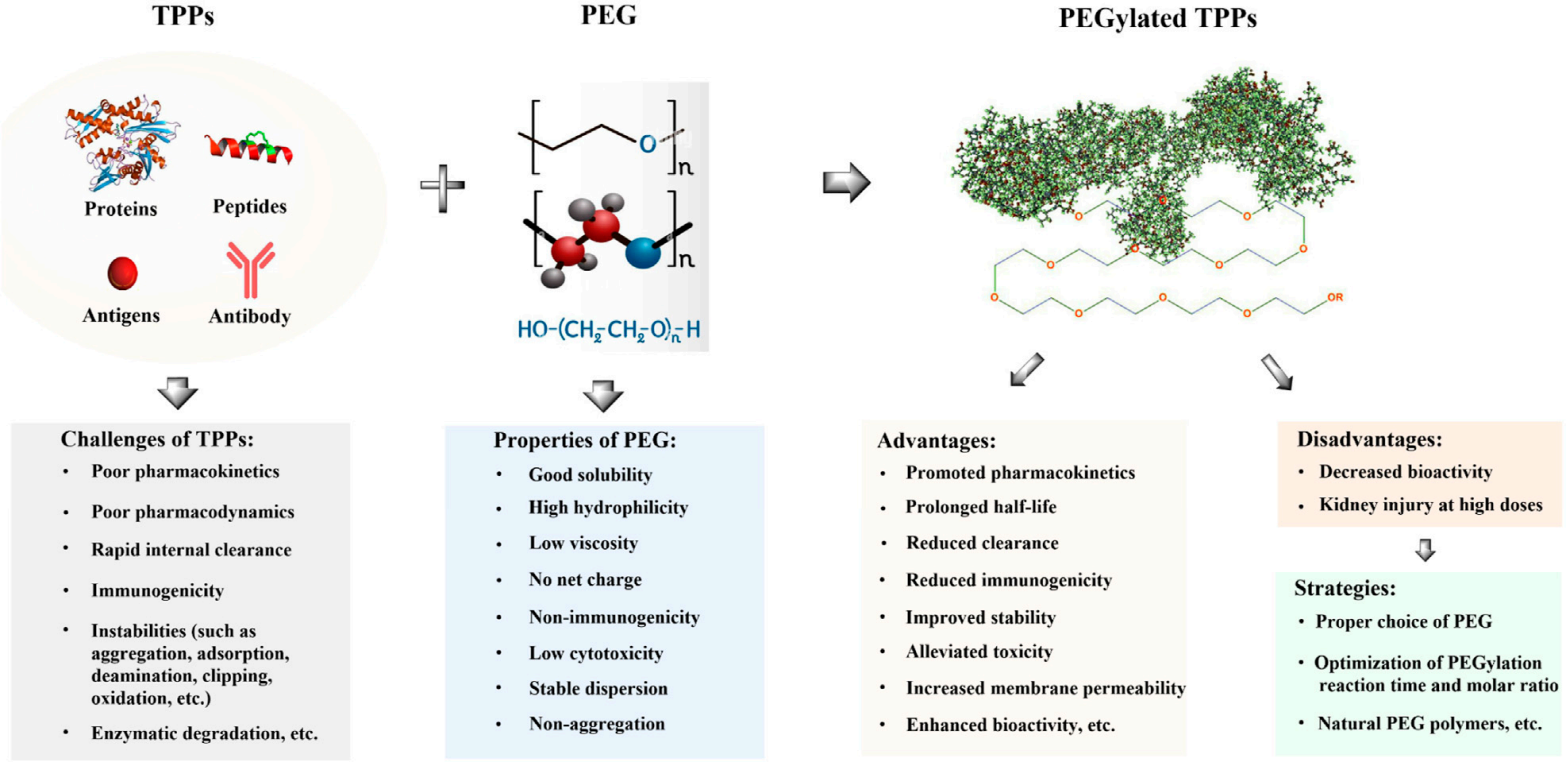
Some advantages and disadvantages of PEGylated TPPs. Challenges in the clinical application of TPPs include their short half-life, poor pharmacokinetics, instabilities, and immunogenicity, which limit their therapeutic effectiveness. PEGylation can enhance the solubility and membrane permeability of TPPs through the hydrophilicity of PEG molecules and improve their pharmacokinetics and efficacy by increasing accumulation in target organs or sites (Levy et al., 1988; Casey et al., 2000; Guiotto et al., 2003; Chae et al., 2009; Singh et al., 2014; Cui et al., 2021; Li et al., 2022). Additionally, PEG modification can reduce clearance by the reticuloendothelial system and immunogenicity by shielding from proteolytic enzymes while decreasing cytotoxicity (Vugmeyster et al., 2012; Benincasa et al., 2015). Moreover, PEGylation of TPPs may promote membrane permeability (Tan et al., 2019; Li et al., 2022). However, a major disadvantage of TPPs is the potential loss of bioactivity or function following PEGylation due to significant conformational changes involved. Subcutaneous administration of PEGylated TPPs may result in low bioavailability due to their waxy nature, and unnatural PEG polymers used can contribute to poor biodegradability. To address these issues, several solutions exist including careful selection appropriate PEG molecules, optimization of reaction conditions for PEGylation (including temperature, time, pH, molar ratio, etc.), and utilization of natural PEG polymers (Witt et al., 2001; Armstrong et al., 2007; Imura et al., 2007b; Schlapschy et al., 2013; Cao et al., 2021; Li et al., 2022).

The linear polymer polyethylene glycol (PEG) is composed of repeated ethylene glycol units [-(O-CH_2_-CH_2_)_n_] and has a molecular weight (MW) range of 0.4–150 kDa. It has been extensively used in the pharmaceutical industry for several decades due to its favorable properties, such as high hydrophilicity, low viscosity, non-immunogenicity, and excellent biocompatibility (Guichard et al., 2017; Zuma et al., 2022a). The covalent conjugation of PEG to TPPs plays a crucial role in regulating and determining the structure, function, activity, immunogenicity, and pharmacokinetic profiles of drug molecules. In the late 1970s, PEG was initially employed to modify bovine serum albumin (BSA), resulting in improved immunological and soluble properties compared to its unmodified form (Abuchowski et al., 1977). In the subsequent two or three decades, PEG modification technology has undergone rapid development to prolong the biological half-life of TPPs, diminish their immunogenicity, and promote their stability, therapeutic efficacy, as well as accumulation in target organs or cells through improved permeability and retention effects (Knauf et al., 1988; Nakaoka et al., 1997; Chapman, 2002; Basu et al., 2006; Treetharnmathurot et al., 2008; Nojima et al., 2009b; Park et al., 2010; Erak et al., 2018; Guichard et al., 2018). It has been demonstrated that PEGylated interferon λ (PEG-IFN-λ) exhibits no inflammatory side effects or broad-spectrum antiviral activity both *in vitro* or *in vivo*, including against hepatitis and symptomatic coronavirus disease 2019 (COVID-19) (Leader et al., 2008; Manning et al., 2010; Du et al., 2015). Reis et al. evaluated the effectiveness of PEG-IFN-λ in preventing COVID-19 and reported a 41% reduction in time to COVID-19-related deaths or hospitalizations following administration of a single subcutaneous injection containing 180 μg of PEG-IFN-λ (Manning et al., 2010). Currently, PEG-IFN-λ1 is the sole available IFN-λ therapeutic agent. The use of PEGylated IFN-λ significantly decreases viral loads among patients with acute COVID-19, and it may serve as an effective therapeutic agent against this disease (Reis et al., 2023). Moreover, pegilodecakin (PEGylated interleukin-10) has been demonstrated to play a pivotal role in the inhibition of tumor growth and metastasis (Autio and Oft, 2019; Tannir et al., 2021). Recently, several PEGylated antimicrobial peptides (AMPs), such as OM19r-8, N6, Onc72, SAAP-148, etc., have shown potential in enhancing resistance against proteolytic enzymes, promoting antibacterial/immunomodulatory activities, and prolonging *in vivo* half-time (Cui et al., 2021; Li et al., 2022; Mohammed et al., 2023; Van Gent et al., 2023). In general, PEGylation of TPPs offers various advantages for overall efficacy. These include improving solubility, stability, permeability, and pharmacokinetic properties, while reducing glomerular filtration clearance rate, immunogenicity, and toxicity. Additionally, it extends drug circulation time (Figure 1) (Knauf et al., 1988; Nakaoka et al., 1997; Chapman, 2002; Basu et al., 2006; Nojima et al., 2009b; Park et al., 2010; Li et al., 2022). However, the widespread application of PEGylated TPPs also presents certain potential drawbacks, such as diminished bioactivity, poor bioavailability and limited biodegradability due to waxy behavior or the use of unnatural PEG polymers (Figure 1) (Witt et al., 2001; Imura et al., 2007a; Armstrong et al., 2007; Schlapschy et al., 2013; Ko and Maynard, 2018; Cao et al., 2021; Li et al., 2022).

Several studies have comprehensively reviewed various aspects of PEGylation, focusing on the technology and modification of non-TPP drugs. In this study, we conducted a comprehensive survey on PEGylated TPPs, providing detailed insights into the physicochemical properties and classification of PEG and its derivatives, sites modified by PEGylation in TPPs, as well as factors influencing the PEG modification process. Furthermore, we highlighted typical cases and current developments in the field of PEGylated TPPs, followed by an analysis of the potential application of this technology in TPPs.

## 2 Physicochemical properties and classification of PEG and its derivatives

### 2.1 Chemical structure of PEG and its derivatives

**FIGURE 2:**
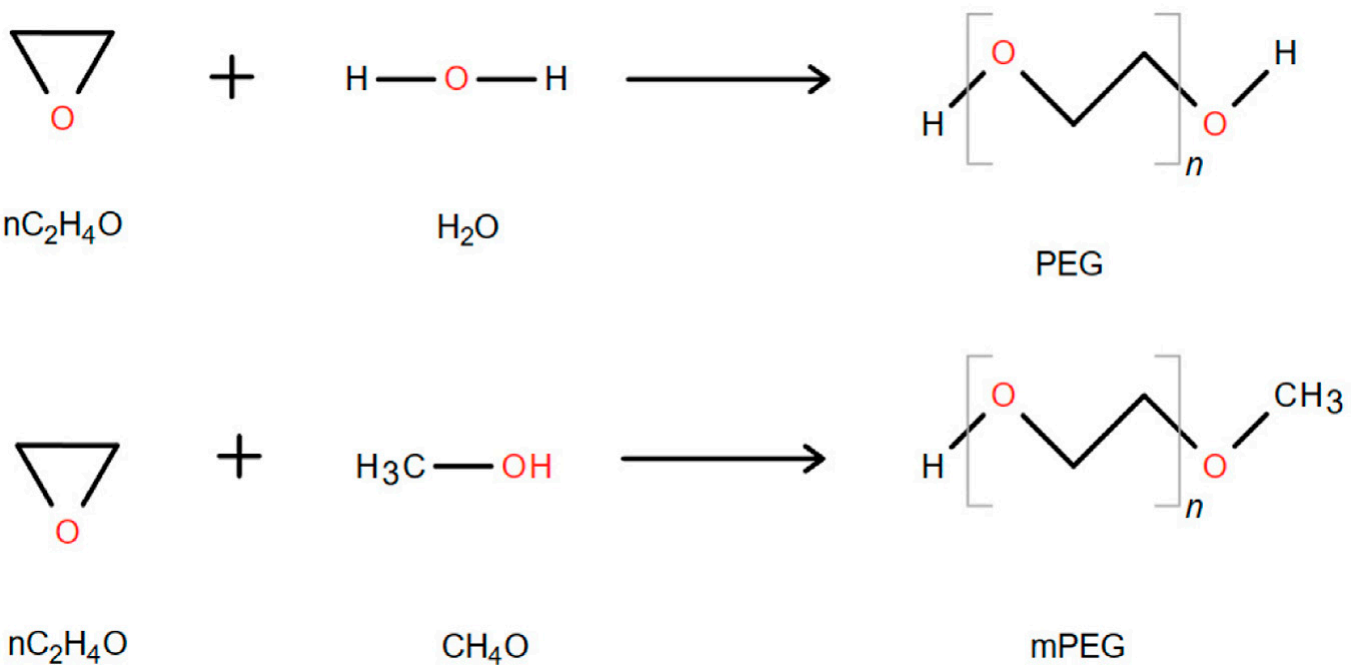
Synthetic chemical formula of PEG and monomethoxy PEG. “n” represents the average number of repeating oxyethylene groups (Zhang et al., 2007).

PEG is a linear polymer with hydroxyl groups at both ends, which is formed by gradually adding ethylene oxide to water or ethylene glycol. It consists of repeated oxyethylene units. The simplest structure of PEG is a straight-chain hydroxyl-terminated polyether represented by the following structural formula: HO-(CH_2_CH_2_O)n-CH_2_CH_2_-OH (Roberts et al., 2002) (Figure 2). The terminal hydroxyl group of PEG serves as the functional group in chemical modification reactions, but its reactivity is low; therefore, it needs to be activated for modifying TPPs. Monomethoxy PEG (mPEG) is commonly used for TPP modification, and its general structure can be depicted as CH_3_O–(CH_2_CH_2_O)n-CH_2_CH_2_-OH (Figure 2). mPEG is obtained by blocking one end hydroxyl group of PEG with a methyl group to prevent crosslinking and agglomeration with TPPs during the modification process (Zhang et al., 2007).

### 2.2 Biological properties of PEG and its derivatives

The MWs of linear and branched chain PEGs used in TPPs typically range from 3 to 60 kDa, which is larger compared to nanomedicines like Doxil and mRNA-based COVID-19 vaccines that have a size of 2 kDa (Na et al., 2008; Shi et al., 2022). PEG exhibits hydration in aqueous solution, with each ethane oxide unit (-CH_2_-CH_2_-O-) binding 2–3 water molecules. Consequently, the apparent MW of PEG in solution is significantly higher than that of proteins or peptides with the same MW (Antonsen and Hoffman, 1992). Youn et al. (2008) used PEGylated salmon calcitonin (sCT) with a relative MW of 5 kDa and observed that the MW of sCT modified with PEG5000 reached as high as 259 kDa, for exceeding its actual MW of 84 kDa. This property not only prolongs the circulating half-life of PEGylated protein drugs but also enhances their stability in solution. Moreover, PEG demonstrates good solubility in water and most organic solvents while being insoluble in ether and aliphatic hydrocarbons, making it an amphiphilic molecule (Henning, 2002). Interestingly, the immunogenicity of PEGylated TPPs may be reduced due to steric hindrance caused by PEG, which can impede immune recognition (Harris et al., 2001; Shi et al., 2022). For instance, PEGylation of asparaginase could eliminate its antigenicity (Abuchowski et al., 1977; Duval et al., 2002; Molineux, 2003; Wang et al., 2012).

### 2.3 Classification of PEG derivatives

**FIGURE 3:**
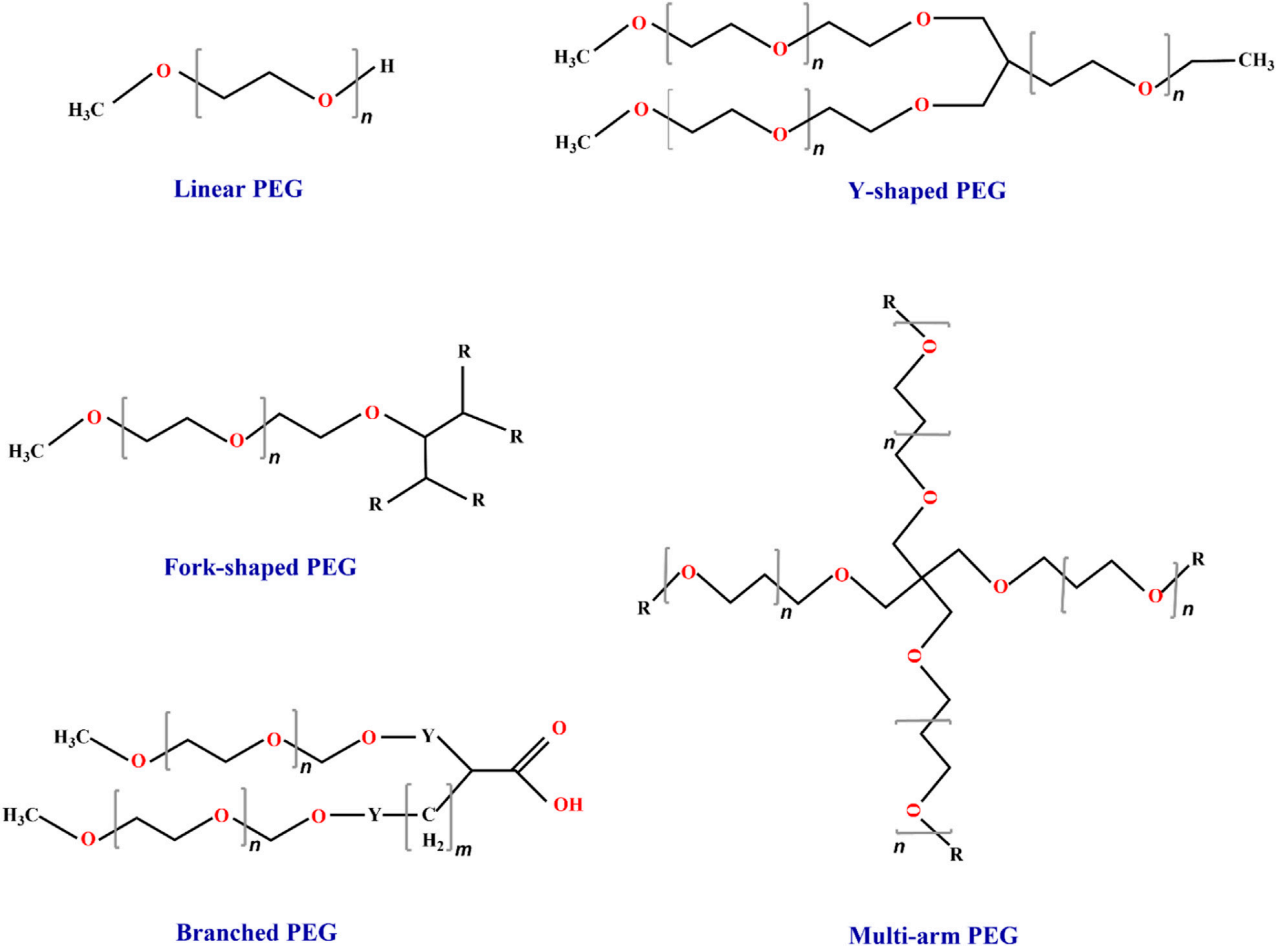
Classification of PEG and its derivatives. The market currently utilizes five types of PEG materials, namely, linear, Y-shaped, multiarm, fork-shaped, and branched materials. Among these options, linear PEG is commonly employed for the PEGylation of TPPs (Kolate et al., 2014; Lu and Zhang, 2018). “n” represents the average number of repeating oxyethylene groups.

With the development of biotechnology, PEG derivatives have been categorized into three distinct generations based on their MWs (Table 1; Figure 3). The first-generation of low MW PEG derivatives primarily involved coupling with the amino group of TPPs (Miron and Wilchek, 1993). Notable examples from this first generation include PEG succinimide carbonate (PEG-SC), PEG benzotriazole carbonate (PEG-BTC), PEG dichlorotriazine, PEG tresylate, PEG p-nitrophenyl carbonate, and PEG trichlorophen. Among these, both PEG-SC (Zalipsky et al., 1992) and PEG-BTC are extensively employed in TPPs (Dolence et al., 1997) as they selectively react with lysine residues within protein molecules to form carbamates. Additionally, Adagen (PEG-ademase bovine), OncoSpar (PEG-aspargase), and PEG-Intron (PEG-interferon α-2b) were also modified using linear PEG from the first generation (Veronese and Pasut, 2005). However, first-generation PEG derivatives often exhibit weak interactions upon conjugation with proteins and undergo side reactions with protein drugs. Consequently, numerous issues arise, including the formation of multiple side reaction products (Abuchowski et al., 1977) and degradation products (Gais and Ruppert, 1995), as well as difficulties in separating modified products (Zalipsky et al., 1992). Furthermore, these derivatives display poor stability, high toxicity, and inadequate homogeneity. In contrast, second-generation PEG derivatives employ more efficient functional groups such as aldehydes, esters, and amides for specific and functional chemical modifications (Wang et al., 2011). For instance, Kinstler et al. (1996) initially discovered PEG-aldehyde derivatives to achieve site-specific modification of the N-terminal amino group of polypeptides. Second-generation PEG derivatives can also accomplish targeted modifications of sulfhydryl groups (Brocchini et al., 2008); commonly used sulfhydryl-modified PEG derivatives include PEG-maleimide (MAL-PEG), PEG-vinyl sulfone (VS-PEG), etc. (Pasut and Veronese, 2012). As the field of PEGylation chemistry advances further, there is a growing demand for heterobifunctional PEG derivatives. Yoo et al. (2012) conjugated folate (FA) to one end of PEG and superparamagnetic iron oxide nanoparticles (SPIONs) to the other end, resulting in the formation of FA-PEG-SPIONs. The introduction of biocompatible PEG into the drug mixture conferred excellent biocompatibility on FA-PEG-SPIONs, including low cytotoxicity, stable dispersion, non-aggregation, and strong optical imaging ability in a mouse model of lung cancer. The third generation of PEG derivatives was developed as branched PEG derivatives, such as tree-type PEG, Y-type PEG, and comb-type PEG, etc.) (Figure 3) (Zhang et al., 2012). Vugmeyster et al. (2012) performed site-specific modification of the antitumor necrosis factor alpha (TNF-α) nano antibody using linear, Y-shaped, and tree-shaped PEG, respectively. Among them, the two-arms branching structure exhibited the longest half-life in rats.

**TABLE 1:** Characteristics of PEG and its derivatives used in TPPs.

Classification	Features	Cases	References
The first generation	Instability, strong toxicity, poor homogeneity, etc.	PEG-dichlorotriazine derivatives, PEG-trifluoroethyl sulfonate, PEG-succinimide succinate, PEG-succinimide carbonate, etc.	Abuchowski et al. (1977), Zalipsky and Lee (1992), Miron and Wilchek (1993), Gais and Ruppert (1995), Dolence et al. (1997)
				The second generation	Better protection the active sites of TPPs	N-terminal site-modified PEG derivatives, such as mPEG-propionaldehyde; mercap to site-modified PEG derivatives, such as PEG-maleimide (MAL-PEG); controlled release PEG derivatives: such as PCLA-PEG-PCLA; heterobifunctional PEG derivatives: such as FA-PEG-SPIONs	Kinstler et al. (1996), Brocchini et al. (2008), Kizilel et al. (2010); Wang et al. (2011), Pasut and Veronese (2012)
The third generation	Higher stability and longer half-life of PEG-modified TPPs	Y-type and tree-type MAL-PEG, etc.	Zhang et al. (2012), Vugmeyster et al. (2012)				

## 3 PEG-modified sites on TPPs

**FIGURE 4:**
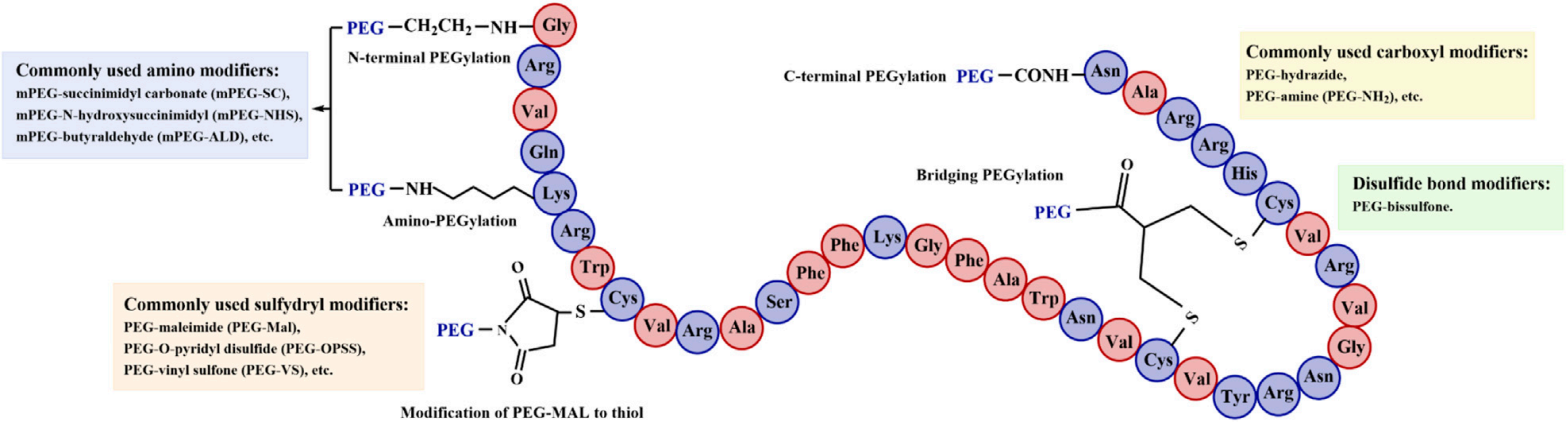
PEG-modified sites in TPPs. The modification reactions can be categorized into acylation, alkylation, redox, and aromatic ring substitution. Theses reactions involve N-terminal PEGylation, C-terminal PEGylation, amino PEGylation, sulfhydryl PEGylation, and bridging PEGylation (Herman et al., 1995; Veronese, 2001; Pasut and Veronese, 2012).

PEG can be covalently conjugated to specific sites in protein drugs, and the common modification sites of TPPs are illustrated in Figure 4 (Pasut and Veronese, 2012). Different activated PEGs were chosen for various modified groups. The modification reactions involving PEG and TPPs exhibit distinct properties, including acylation, alkylation, redox, and aromatic ring substitution. PEG chemically modifies side chain groups such as amino, sulfhydryl and carboxyl groups of TPPs (Herman et al., 1995; Veronese, 2001). This review discusses the methodologies employed for amino, sulfhydryl, and carboxyl group modifications in PEG-conjugated TPPs along with their advantages and disadvantages (Table 2).

**TABLE 2:** Comparison of three major PEG modification methods used in TPPs.

Method	Modification site	Advantage	Disadvantage	References
Site-modified amino	N-Terminal amino group and amino groups on lysine residues on the molecular surface	It is the most frequently modified group in the chemical modification of TPPs, and has higher nucleophilic reactivity	There are many free amino groups in the protein molecules, which are randomly modified, and the modification is easy to cause the loss of protein activity and the heterogeneity of the final product	Gaertner and Offord (1996), Kinstler et al. (2002), Yamamoto et al. (2003), Yoshioka et al. (2004), Youn and Lee (2007), Youn et al. (2007a), Youn et al. (2007b), Zhou et al. (2014)
					Site-modified thiol	Molecular surface cysteine free thiol and disulfide bond	TPPs are usually not high in content, the position is determined, and quantitative and site-specific modifications can be performed	Low modification rate	Hao et al. (2006), Brocchini et al. (2006), Shaunak et al. (2006), Zloh et al. (2007), Balan et al. (2007), Chi et al. (2008), Brocchini et al. (2008), Zhao et al. (2012)
Site-modified carboxyl	Glutamic acid, aspartic acid and terminal carboxyl group	Mild reaction	It is easy to produce other cross-linking reactions	Gault et al. (2008), Veronese (2001)					

### 3.1 Site-modified amino

The groups involved in the PEGylation of TPPs typically consist of α- and ε-amino groups. Therefore, the PEGylation of amino groups in TPPs can be categorized into N-terminal α-NH_2_ modification and lysine ε-NH_2_ modification. Lysine is one of the most prevalent amino acids in TPPs, usually located on the surface of protein molecules’ three-dimensional structure, making it easily accessible for conjugation with PEG modifiers. A unique two-step site-specific PEGylation method was employed to produce Lys^21^-amine PEGylated growth hormone-releasing factor (GRF) (1–29) through a newly devised site-specific PEGylation process using a 9-fluorenylmethoxycarbonyl (FMOC)-protection/deprotection method at N-α Tyr^1^ and Lys^12^ (Youn and Lee, 2007). The His^7^-(N-terminus), Lys^26^-or Lys^34^-amine specific PEGylation of glucagon-like peptide-1 (GLP-1) was prepared by a site-specific PEGylation process using a maleic anhydride (MA)-protection/deprotection method (Youn et al., 2007a). Similarly, Lys^18^-, Cys^1^-or Lys^11^-amine mono-PEGylated salmon calcitonin (sCT) was generated using an FMOC protection/deprotection technique (Youn et al., 2007b). This strategy resulted in higher production yield, longer half-lives, and improved biological stability compared to conventional non-specific PEGylation (Youn et al., 2007a; Youn et al., 2007b; Youn and Lee, 2007). Regarding the N-terminal α-NH_2_ modification, the pKa of the protein’s N-terminal α-NH_2_ is typically around 7.6 to 8.0, while the pKa of lysine ε-NH_2_ is generally in the range of 10.0–10.2. Therefore, precise modification of the N-terminal α-NH_2_ can be achieved by controlling pH under acidic conditions (Kinstler et al., 2002). Yamamoto et al. (2003) and Yoshioka et al. (2004), Yoshioka et al. (2011) proposed a novel method for site-specific PEGylation at fixed points on TPPs that lack lysine residues entirely, such as TNF-α. These Lys-deficient TPPs were mono-PEGylated exclusively at their N termini and exhibited enhanced bioactivity *in vitro* as well as superior antitumor therapeutic efficacy compared to randomly mono-PEGylated wild-type counterparts. Additionally, when a peptide or protein has serine or threonine at its N-terminus, periodic acid can be used to oxidize the functional group into an aldehyde group, enabling subsequent site-directed modification with PEG-hydrazide (Gaertner and Offord, 1996; Zhou et al., 2014).

### 3.2 Site-modified thiol

Modification of the cysteine-free sulfhydryl group or disulfide bond on the protein surface is a method employed to achieve targeted modifications. Thiol modification of cysteine refers to the introduction of PEG fixed-point modification on proteins containing a single cysteine residue. Examples of site-directed PEG modifications include Cys34 in human serum albumin (HSA) and Cys17 in granulocyte colony-stimulating factor (G-CSF) (Hao et al., 2006; Zhao et al., 2012). For peptides or proteins lacking sulfhydryl groups, site-specific modifications can be achieved through genetic engineering by introducing sulfhydryl groups at appropriate locations. Chi et al. (2008) employed a microwave-assisted solid-phase synthesis technique to synthesize GLP-1 (7–36), wherein Gly and Cys were substituted for Ala at positions 8 and 30, respectively, followed by PEG-modification of Cys at position 30. In the context of disulfide bond modification, the presence of a disulfide bond in a peptide or protein plays a crucial role in stabilizing its secondary and tertiary structure; however, modifying this bond may potentially lead to loss of activity. Hence, the development of highly efficient PEG modifiers and feasible strategies for site-specific disulfide bond modification has become pivotal. Brocchini et al. (2006) devised a dialkylated PEG reagent capable of alkylating both thiols within the reduced disulfide bond to form a three-carbon bridge. By employing such a three-carbon bridge, PEG can be covalently attached to proteins while preserving their biological activity of TPPs (Balan et al., 2007).

### 3.3 Site-modified carboxyl groups

The side chains of TPPs contain a significant number of carboxyl groups, with modification sites including glutamic acid, aspartic acid, and carboxyl groups at the end. In recent years, mPEG-hydrazine has emerged as a specific modifier capable of binding to carboxyl groups. In the presence of 1-ethyl-3-dimethylaminopropylcarbodiimide hydrochloride (EDC) or dicyclohexylcarbodiimide (DCCI), the carboxyl group can form an amide bond with the amino group of mPEG-NH_2_ (Gault et al., 2008). Additionally, in acidic conditions (pH 4.5–5.0) and in the presence of EDC, protonation occurs without any cross-linking reactions involving the amino group in proteins or peptides (Veronese, 2001).

### 3.4 Site-specific modifications of the other groups

Other groups on TPPs can also be utilized as potential sites for PEGylation, such as amino acid residues located at the protein or peptide’s C-terminus (Cazalis et al., 2004), glycosylation sites consisting of serine and threonine residues (Giorgi et al., 2014), and histidine residue (Wylie et al., 2001; Zuma et al., 2022b; Zuma et al., 2022a; Khataminezhad et al., 2023). Both IFN α-2b and IFN β-1b were fused to the *Mycobacterium xenopi* GyrA intein and expressed in *Escherichia coli*. They were subsequently cleaved by hydrazine to generate the corresponding IFN α-2b C-terminal hydrazide, followed by site-specific modification with a pyruvyl derivative of PEG with a MW of 10 kDa (Thom et al., 2011). The PEGylated forms of IFN α-2b and IFN β-1b exhibited excellent antiviral activity, indicating that intein technology is compatible with TPPs containing disulfide bonds and can be used for C-terminal PEGylation of other TPPs, including antibody fragments, using PEGs with different MWs. Both domain antibody (dAb) and IFN α-2a tagged with His tags at the C-terminus and the N-terminus underwent site-specific PEGylation at their histidine tags, resulting in extended circulation half-lives (Cong et al., 2012).

Additionally, genetic engineering and other techniques have been employed to introduce specific groups at the C-terminus or N-terminus of proteins or peptides for site-mediated PEGylation using histidine tags (Thom et al., 2011; Cong et al., 2012). Moreover, noncanonical amino acids have also been incorporated into recombinant TPPs for targeted PEGylation (Nischan and Hackenberger, 2014).

## 4 Factors affecting PEGylation of TPPs

The reaction of PEGylated TPPs can be influenced by various factors, including the selection of an appropriate PEG modifier, the molar ratio between PEG and protein drugs, the optimization of reaction conditions (such as temperature, pH, and time), the MW distribution of PEG, and the structural characteristics of its molecular chains.

### 4.1 Selection of PEG modifiers

The selection of PEG modifiers should take into account the relative molecular mass (Mn), MW distribution coefficient (PDI), modification sites, functional groups, and molecular chain structure. Previous studies have demonstrated that the reaction time of modified protein drugs is directly proportional to the amount of PEG and Mn conjugated, while the biological activity is inversely proportional to this amount (Chiu et al., 2010; Ma et al., 2013; Morgenstern et al., 2017). However, excessive Mn in PEG-modified protein drugs may lead to a decrease in their overall biological activity (Weinberg et al., 2018). Moreover, it is preferable for PEGylated agents to have a small PDI and a narrower MW distribution as this facilitates separation and purification of PEG-modified TPPs (Chen et al., 2018). When selecting the modification site, it is important to consider the structure‒activity relationship analysis of TPPs. Preferably, residues on the protein surface that do not bind to receptors should be chosen as modification sites for modified TPPs to retain their high biological activity (Dozier and Distefano, 2015).

For the specificity of the modification reaction, it is crucial to select and utilize PEG modifiers with appropriate functional groups, such as cross-functionalized mono-sulfone PEG, in TPPs (Pasut and Veronese, 2012). It should be noted that PEGylated molecules with distinct chain structures of linear and branched chains have various biological characteristics that can affect numerous pharmacokinetic parameters of TPPs (Vugmeyster et al., 2012; Zhang et al., 2012). Branched PEG modifiers exhibit reduced accessibility to hidden sites and enhanced stability against proteolysis compared to linear PEG modifiers (Wan et al., 2017; Sun et al., 2023).

### 4.2 Molar ratio of PEG to TPPs

Generally, PEG molecules with lower MWs and degrees of PEGylation may lead to higher residual activities of TPPs, while higher PEG MWs and degrees of PEGylation can enhance the conformational and colloidal stability of TPPs (Morgenstern et al., 2017). The increase in the molar ratio of PEG to TPPs, results in elevated relative MWs of the PEG-modified TPPs and modification rate, which affect the biological activity of the TPPs. Behi et al. (2018) conducted modifications on rhG-CSF using different molar ratios of methoxy PEG propionaldehyde (mPEG-ALD) and recombinant human granulocyte colony stimulating factor (rhG-CSF), finding that a molar ratio of 5:1 for mPEG-ALD to protein yielded optimal single-PEGylated rhG-CSF. Chinol et al. (1998) modified avidin (AV) with monomethoxy PEG (mPEG) at various molar ratios, observing a gradual decrease in AV-biotin binding rate as more mPEG was attached to AV. BSA modified with PEG800 exhibited maximum conformational stability at a BSA:PEG molar ratio of 1:0.75 due to surface residue protection and buried hydrophobic residue shielding by PEG (Rawat et al., 2020). Therefore, it is crucial to control the appropriate proportion between PEG and TPPs during modification processes (de Lencastre Novaes et al., 2010).

### 4.3 Reaction pH and time and TPPs’ concentration

The pH is a critical factor that can influence the modification of PEG-modified TPPs. By controlling the appropriate pH, specific modifications can be made to the amino acid residues in the protein, thereby enhancing modification specificity and reducing separation difficulties. Lactoferrin was modified with N-hydroxysuccinimide-activated PEG (PEG-NHS) to enhance its pharmacokinetic properties (Nojima et al., 2009a). The results demonstrated that pH played a crucial role in achieving optimal conditions for PEG-NHS-modified bovine lactoferrin. While increasing reaction time led to higher modification rates of PEGylated products, it also resulted in increased heterogeneity of the modified products. Laccase was subjected to monomethoxy PEG (20, 30, 40 kDa and 40 kDa-branched) modification durations of 4 h and 17 h, respectively (Mayolo-Deloisa et al., 2015). The optimal reaction time for laccase PEGylation was found to be 4 h at a PEG:protein molar ratio of 4:1; furthermore, laccases modified with 30 kDa linear PEG exhibited greater activity compared to other types of PEGs (Mayolo-Deloisa et al., 2015).

Additionally, the concentration of TPPs plays a crucial role in PEGylation reactions. Koussoroplis et al. (2013) reported that higher concentrations of recombinant human deoxyribonuclease I (rhDNase) result in more efficient PEGylation reactions. When the concentration of rhDNase was 1 mg/mL, and the molar ratio of PEG to protein was 16:1, the reaction time lasted for 96 h (Guichard et al., 2017). However, increasing the concentration of rhDNase to 10 mg/mL resulted in a reduced molar ratio of PEG to protein to 4:1 and a shortened reaction time with overnight incubation (Mahri et al., 2021).

### 4.4 MWs and molecular chain structures of PEG

Increasing the MW of PEG can extend the half-life of a drug, and branched PEG offers more advantages compared to linear PEG. Higher MWs of branched PEG exhibit a stronger steric hindrance effect, thereby reducing the likelihood of accessing the active site. Additionally, binding of branched PEG to the surface of TPPs effectively screens for surface antigens and enzymatic hydrolysis sites while minimizing immunogenicity (Pasut and Veronese, 2007). Yoshioka et al. (2004) utilized linear PEG with MWs of 5 and 20 kD as well as branched PEG with MWs of 10 and 40 kDa to modify lysine-deficient TNF-α. The *in vitro* activity retention rates were found to be 82%, 58%, 93%, and 65% respectively. Lysine-deficient TNF-α modified with linear PEG at a MW of 20 kDa and branched PEG at a MW of 10 kDa exhibited higher antitumor activity in mice compared to linear PEG at a MW of 5 kDa; however, modification with branched PEG at a MW of 40 kDa resulted in loss of activity (Yoshioka et al., 2004). Furthermore, increasing the MW of PEG leads to enhanced half-life for modified TPPs *in vivo* when compared to unmodified TPPs. rhDNase was conjugated with linear 20 kDa, linear 30 kDa or 2-armed 40 kDa PEG. While PEG20-rhDNase and PEG30-rhDNase progressively lost their activity over time in the lungs of mice, PEG40-rhDNase remained active. Notably, PEG20-rhDNase lost most of its activity. Additionally, compared to PEG20-rhDNase and PEG30-rhDNase, PEG40-rhDNase had lower thermodynamic stability (Guichard et al., 2017; Mahri et al., 2023). Similar destabilization upon PEGylation was also observed in cytochrome C modified with a 5 kDa PEG and recombinant human interleukin-1 receptor antagonist (rhIL-1ra) modified with a 20 kDa PEG, where TPPs’ thermodynamic stability decreased due to the modification by the polymer (García-Arellano et al., 2002; Sorret et al., 2019). The thermal stability of PEGylated green fluorescent protein (GFP) was assessed by de Lencastre Novaes et al. (2010) at temperatures ranging from 70°C to 95°C, considering different molar masses and concentrations of PEG. It was found that only PEG with molar masses of 600 and 4,000 g/mol provided protection to GFP molecules at 75°C, while the stabilization effect was not observed for GFP when using PEG with a molar mass of 10,000 g/mol between temperatures of 75°C and 95°C.

## 5 Typical cases of PEGylated TPPs

Among PEG-modified TPPs, several promising PEG-modified TPPs are highlighted in this study.

### 5.1 PEGylated AMPs

AMPs, also known as host defense peptides (HDPs), play a crucial role in innate immunity in multicellular organisms (Yang et al., 2017). Due to their broad antimicrobial spectrum and low toxicity, AMPs have emerged as potential candidates for novel therapeutic agents (Zasloff, 2002; Wang et al., 2020). Over the last three decades of AMP research, these peptide molecules have been proven to possess multiple biological functions including antibacterial, antifungal, antiviral, antiparasitic, anticancer and immunomodulatory activities (Mahlapuu et al., 2016; Mazurkiewicz-Pisarek et al., 2023). However, the clinical application of many AMPs is limited to topical medications only due to issues such as instability, potential antigenicity, rapid renal clearance, short circulation half-life and low therapeutic indices *in vivo* (Darveau et al., 1991; Steinberg et al., 1997; Zasloff, 2002; Harris and Chess, 2003; Chan et al., 2006; Hancock and Sahl, 2006; Răileanu et al., 2023). Furthermore, some AMPs exhibit reduced activity when exposed to serum (Johansson et al., 1998; Yeaman et al., 2002), plasma (Yeaman et al., 2002) or divalent cations (Matsuzaki et al., 1997; Johansson et al., 1998).

To address the limitations of AMPs, researchers have employed various strategies. These include targeted mutation techniques (Braman, 2002), fusion expression with proteins like albumin and immunoglobulin that possess longer half-lives (Sung et al., 2003; Qin et al., 2005), and chemical modifications to alter the structure of AMPs (Veronese and Mero, 2008). Among these approaches, PEG-modified AMPs have garnered significant attention due to the favorable biocompatibility of PEG. For instance, PEGylation substantially enhanced the enzymatic resistance of nisin A (34 aa) (Guiotto et al., 2003). Singh et al. (2014) demonstrated that PEGylation effectively reduced toxicity, increased selectivity, maintained anti-inflammatory effects, and decreased serum protein clearance of KYE28 peptide. Similarly, other AMPs such as M33, MA, and SAAP-148 exhibited improved stability and selectivity after being modified with different MWs of PEG. Additionally, they showed reduced scavenging by serum proteins while retaining their anti-inflammatory activity and promoting antimicrobial activity following PEGylation (Table 3) (Zhang et al., 2008; Falciani et al., 2014; Van Gent et al., 2023). Compared to the unmodified peptide, C-terminal PEGylated N6 displayed broader biodistribution in mice along with slower renal clearance and prolonged *in vivo* half-life (Li et al., 2022).

**TABLE 3:** Typical cases of PEGylated TPPs.

Name	Modification position	Molecular mass of PEG (Da)	Improved performance	References
Peptide drugs				
M33	The C-terminus of the three lysine-branching cores	175	Improved stability of elastase against *Pseudomonas aeruginosa*	Falciani et al. (2014)
KYE28	N- and C-terminus	200, 600, 1,100 and 2,200	The combination of reduced toxicity, increased selectivity, and retained anti-inflammatory effect after PEGylation, as well as reduced scavenging by serum proteins	Singh et al. (2014)
CaLL	N-terminus	671.4 and 1,007.3	Reduced cytotoxicity of CaLL; airway administration of PEG-CaLL did not disrupt the lung epithelial barrier, whereas CaLL caused pulmonary edema	Morris et al. (2012)
Nisin A	C-terminus and the α-amino group	5,000	PEGylation improves its solubility and protect it toward enzymes present in nonoptimally processed food	Guiotto et al. (2003)
MA	C-terminus	750 and 1,100	The stability of PEGylated peptides was significantly enhanced in the presence of chymotrypsin and serum; the antibacterial activity of PEGylated peptides in serum was significantly enhanced	Zhang et al. (2008)
Tachyplesin I	N-terminus	5,000	PEGylation significantly reduced cytotoxicity	Imura et al. (2007a)
Magainin 2	N-terminus	5,000	PEGylation significantly reduces cytotoxicity and the antimicrobial activity at the same time	Imura et al. (2007b)
73c	Cysteine residue	2,000	PEGylation significantly alleviates toxicity toward human cells and reduced aggregation	Kumar et al. (2019)
Bac7(1–35)	Amide or ester bond C-terminal PEGylation of Bac7(1–35)	20,000	PEGylation allows the peptide to have a wide distribution in mice, and a slow renal clearance	Benincasa et al. (2015)
CPP	Covalently bonded on the surface of CPP	4,000 and 10,000	PEGylation promoted cellular uptake and pharmacodynamics	Tan et al. (2019)
OM19r-8	N-terminus	5,000	PEGylation prolonged the half-life of OM19r-8	Cui et al. (2021)
N6	N-terminus, C-terminus, and Cys residues	145–1,127	PEGylated N6 at the C-terminus improved its proteolytic stability, antibacterial and anti-inflammatory activity, and prolonged *in vivo* half-time; PEGylated N6 at the N-terminus and Cys residues reduced its antibacterial activity	Li et al. (2022)
Onc72	C-terminus	5,000–20,000	PEGylation improved proteolytic stability and reduced hemolytic activity	Mohammed et al. (2023)
SAAP-148	C-terminus	201.6–1,302.9	PEGylation improved antibacterial selectivity index and immunomodulatory activities of SAAP-148	Van Gent et al. (2023)
Polypeptide L-P(EG3Glu)	N-/C-terminus	20,000–40,000	PEGylation reduced immunogenicity and increased safety in rats	Sun et al. (2023)
Antibodies				
F9	NN	5,000	PEGylated F9 enhanced accumulation in tumors, improved tumor specificity, and altered the pharmacokinetics	Delgado et al. (1996)
DFM (cross-linked di-Fab' of A5B7)	NN	5,000 and 25,000	PEGylated DFM significantly increased the circulating half-life and tumor uptake levels	Casey et al. (2000)
mCC49 Fab'	Site-specific (Cys) attachment of three branched Mal-dPEG at Fab'	1,600–2,691	PEGylated Fab' induced AUC increases and slower blood clearance	Ding et al. (2013)
IL-17A F(ab′)2 and IL-13 Fab′	Site-selective thiol (Cys)	40,000	PEGylated IL-17A F(ab′)2 and IL-13 Fab′ prolonged the residence time in the lungs of mice	Koussoroplis et al. (2014)
IL-17A Fab′	Site-selective thiol (Cys)	40,000	PEGylated IL-17 Fab' increased the residence time in the lungs of mice, rats and rabbits	Freches et al. (2017)

NN: no data.

### 5.2 PEGylated interferon

In clinical application, interferon α-2b exhibits a short circulating half-life, limited immunogenicity and antigenicity, as well as rapid clearance by the circulatory system, resulting in significant inconvenience for patients' daily life (Bell et al., 2008). Furthermore, high-dose regimens of interferon α-2b are associated with considerable toxicity (Kirkwood et al., 2002). Conjugation of PEG to therapeutically valuable proteins represents an important and effective strategy to address these challenges and has been extensively employed in TPPs to reduce elimination rate while enhancing systemic exposure without compromising biological activity (Roberts et al., 2002). Several studies have demonstrated that peginterferon α-2b is more efficacious than non-PEGylated interferon in the treatment of hepatitis (Carrat et al., 2004; Khalili et al., 2005), which can be attributed to its altered pharmacokinetic (PK) profile leading to prolonged drug exposure (Zeuzem et al., 2000; Lindsay et al., 2001). Clinical evidence indicates that high-dose PEGylated interferon α-2b significantly reduces disease recurrence in resected stage III melanoma patients compared to unmodified interferon α-2b (Daud et al., 2010). Additionally, PEGylated interferon α-2b has shown effectiveness in treating hepatitis C virus (HCV) among children with end-stage renal disease (ESRD) (Mogahed et al., 2016). Moreover, PEGylation of interferon β-1a has been found to improve its pharmacokinetic and pharmacodynamic properties (Pepinsky et al., 2001; Cocco and Marrosu, 2015). These findings collectively demonstrate that optimal PEG modification enhances the bioavailability of interferons.

### 5.3 PEGylated asparaginase

The bacterial enzyme L-asparaginase, found in Gram-negative bacteria, can inhibit normal protein synthesis in tumor cells by degrading L-asparagine, leading to cell death (Narta et al., 2007). However, due to short half-life and antigenicity, L-asparaginase induces severe allergic reactions (Chung, 2010). PEGylated L-asparaginase has the ability to shield antigenic epitopes, reduce immunogenicity, prolong plasma retention time, and decrease proteolysis and renal excretion (Duval et al., 2002; Wang et al., 2012; Molineux, 2023). Clinical trials of PEGylated L-asparaginase began in 1984 and it was found to be safe for patients who had previously experienced allergic reactions to *E*. *coli* or *Erwinia* L-asparaginase (Graham, 2003). FDA approval for PEGylated asparaginase (Rhone-Poulenc Rorer as Oncaspar^®^) was granted in 1994 for the treatment of acute lymphoblastic leukemia (ALL) patients who are hypersensitive to the two native isoforms of the enzyme (Pasut and Veronese, 2009) (Table 3). Currently, improved PEGylated asparaginase with reduced hyposensitivity and a longer half-life is widely used in pediatric ALL patients (Avramis and Tiwari, 2006; Zhang et al., 2020; Riley et al., 2021). These findings demonstrate that PEGylated asparaginase can effectively reduce immunogenicity while extending its half-life for improved clinical applications.

### 5.4 PEGylated antibodies

Over the past decade, PEGylated antibodies have been extensively reported in the field of tumor immunotherapy (Table 3). PEGylation of antibodies prolonged circulation half-life and reduced immunogenicity when introduced into xenograft models (Casey et al., 2000; Chapman, 2002). Kitamura et al. (1990) evaluated the efficacy of murine monoclonal antibodies A7 (MAb A7) and F(ab′)_2_ fragments modified with PEG (MW of 5,000 Da) both *in vitro* and *in vivo*; their findings demonstrated that PEGylated MAb A7 and F(ab′)_2_ exhibited prolonged half-lives and enhanced accumulation within tumors compared to their unmodified counterparts. Furthermore, it has been shown that PEGylation of F(ab′)_2_ fragment and Fab' fragments derived from the A5B7 antibody targeting carcinoembryonic antigen (CEA), significantly enhances antibody accumulation within tumors while prolonging circulating half-life and reducing immunogenicity. However, no significant advantage was observed for PEGylated immunoglobulin G (IgG) over unmodified forms, indicating that PEGylated antibody fragments may possess an advantage over intact IgG modified with PEG for tumor targeting due to improved tumor penetration capabilities (Pedley et al., 1994). The findings suggest that PEGylated antibody fragments hold promise as effective drug carriers for targeted cancer chemotherapy. Several studies have demonstrated altered biodistribution of antibodies or antibody fragments, such as the Fab' fragment (F9) of A5B7 and mCC49 Fab' after PEG modification, resulting in increased tumor accumulation and reduced levels in normal tissues (Table 3) (Delgado et al., 1996; Chapman, 2002; Ding et al., 2013). Moreover, PEGylation significantly prolongs the local residence time of antibody fragments like anti-interleukin-17A (IL-17A) F(ab′)2 and anti-IL-13 Fab’ greatly in the lungs of rats, mice, and rabbits without causing significant pulmonary toxicity. In contrast, unconjugated IL-17A is cleared from the lungs within 24 h (Koussoroplis et al., 2014; Freches et al., 2017; Freches et al., 2019). Overall, PEGylated antibodies have great potential to revolutionize immunotherapy for chronic diseases.

## 6 Prospects of PEG-modified TPPs

Since Davis’s pioneering research on PEGylated protein drugs in the 1970s, the field of long-acting protein drugs has increasingly focused on PEGylation of TPPs (Davis et al., 1979; Davis, 2002). In 1981, Davis and Abuchowski established Enzon, the first company dedicated to PEGylation, which gained FDA approval in March 1990 for their groundbreaking PEGylated protein drug (Adagen) (Lee et al., 1991). Subsequently, numerous PEGylated TPPs have emerged in research; however, many are still undergoing clinical trials or under development. As research on PEG derivatives and modification technology intensifies, the limitations of non-specific site modification technology used to generate PEGylated TPPs have gradually come to light. Consequently, targeted modifications of PEGylated protein drugs have entered clinical trials. In 2002, Amgen’s pegfilgrastim (trade name: Neulasta^®^), a recombinant human granulocyte colony stimulating factor modified with site-directed PEGylation technology, became one of the most successful and also the first FDA-approved PEGylated protein drug (de Graaf et al., 2009). Pegfilgrastim is a mPEG covalently linked to the N-terminal amino group of rhG-CSF, resulting in an approximately 10-fold increase in its *in vivo* half-life compared to the unmodified form (Kinstler, et al., 1996; Bowen et al., 1999). Cetuzumab (Cimzia^®^) is a protein drug that has been modified with PEG and was introduced into the market in 2008. It represents the first PEGylated anti-TNF antibody, where a 40 kDa PEG moiety was specifically attached to the free cysteine residue at the C-terminus of the Fab' fragment of this humanized monoclonal antibody against TNF-α (Pasut, 2014).

Pegcetacoplan (APL-2/Empaveli) is a PEGylated cyclic peptide that functions as a complement C3 inhibitor. It received FDA approval in 2019 for the treatment of ocular diseases, including age-related macular degeneration (AMD) and paroxysmal nocturnal hemoglobinuria (PNH), based on successful clinical trials (Turecek et al., 2016; Hoy, 2021; Ji et al., 2021; Weitz, 2023). Other PEGylated TPPs, such as α1-antitrypsin, IFN α, and Fab fragments, have demonstrated increased residence time in the lungs and improved stability within the airways (Cantin et al., 2002; Koussoroplis et al., 2014; McLeod et al., 2015; Freches et al., 2017; Patil et al., 2018). These modifications enhance the ocular and pulmonary penetration and retention capabilities of TPPs, resulting in prolonged duration of action and reduced dosage frequency. This approach holds promise for improving patient compliance while minimizing systemic side effects associated with systemic administration of PEGylated TPPs for retinal disorder treatment.

With the advancement of science and technology, progressively more sophisticated PEG-modified TPPs currently in experimental or theoretical stage will gradually transit into clinical trials, thereby expanding the scope of applications and enhancing the development prospects for PEG-modified TPPs. Simultaneously, as the human genome project (HGP) undergoes comprehensive exploration, an increasing number of bioactive TPPs will be unearthed, further establishing PEG modification as a pivotal approach to maximize the efficacy of TPPs. Table 4.

**TABLE 4:** PEGylated TPPs in clinical practice.

Product name	PEG conjugate	Modified situation	Adaptation disease	Approved year	References
Adagen^®^	PEG-bovine adenosine deaminase	Random, single-chain 5 kDa PEG, amino multimodified mixture	Severe combined immunodeficiency disease caused by amide deaminase deficiency (SCID)	1990	Booth and Gaspar (2009)
Oncaspar^®^	PEG-asparaginase	Random, single-chain 5 kDa PEG, amino multimodified mixture	Acute lymphocytic leukemia	1994	Vimal and Kumar (2017)
Doxil	Liposomal	2 kDa	Ovarian cancer, multiple myeloma	1995	Bhowmik et al. (2018)
PegIntron^®^	PEG-interferon-α2b	Random, linear 12 kDa PEG, amino modified	Alone or with ribavirin combination medication, treatment of hepatitis c	2000	Cheng et al. (2014)
Pegasys^®^	PEG-interferon-α2a	Random, branched 40 kDa PEG, amino modified	Alone or with ribavirin combination medication, treatment of hepatitis c	2002	Rajender Reddy et al. (2002); Cooksley (2005)
Neulasta^®^	PEG-G-CSF	Selective, linear 20 kDa PEG, N-terminal modification	Neutropenia	2002	Aapro et al. (2017)
Somavert^®^	PEG-human growth hormone mutein antagonist	Random, linear 5 kDa PEG, amino modified	Acromegaly	2002	Trainer et al. (2000)
Macugen^®^	PEG-anti-VEGF aptamer	Selective, branched 40 kDa PEG, amino modified	Wet age-related macular degeneration	2004	Ng EW et al. (2006)
Mircera^®^	PEG-erytropoietin	Random, linear 30 kDa PEG, amino modified	Anemia associated with chronic kidney disease	2007	Seo et al. (2023)
Cimzia^®^	PEG-anti-TNF Fab'	Selectivity, branched 40 kDa PEG, thiol modification	Rheumatoid arthritis and Crohn’s disease	2008	Punzi et al. (2014)
Krystexxa^®^	PEG-uricase	Random, branched 10 kDa PEG, amino modified	Chronic gout	2010	Sherman et al. (2008); Schlesinger and Lipsky (2020)
Asclera	Dodecyl alcohol	400 Da	Varicose veins	2010	Rabe et al. (2021)
Sylatron	Peginterferon-alfa-2b	12 kDa	Melanoma	2011	Cheng et al. (2014)
Omontys^®^	PEG-erythropoiesis stimulant	Selective, branched 40 kDa PEG, amino modified	Anemia in patients with chronic kidney disease undergoing dialysis	2012	Fishbane et al. (2013)
Plegridy	Peginterferon beta-1a	20 kDa	Multiple sclerosis	2014	Hoy (2015)
Movantik	Naloxone	339 Da	Constipation	2014	Floettmann et al. (2017)
Adynovate	Recombinant antihemophilic factor	≥1 × 20 kDa	Hemophilia A	2015	Walsh and Walsh (2022)
Jivi	Recombinant antihemophilic factor	2 × 30 kDa	Hemophilia A	2017	Gogia et al. (2023)
Rebinyn	Recombinant coagulation factor lX	40 kDa	Hemophilia B	2017	Syed Y Y (2017)
Udenyca	G-CSF	20 kDa	Infection during chemotherapy	2018	Brokx et al. (2017)
Palynziq	Recombinant phenylalanine ammonia lyase	∼9 × 20 kDa	Phenylketonuria	2018	Longo et al. (2019)
Revcovi	Recombinant adenosine deaminase	80 kDa	ADA-SCID	2018	Bradford et al. (2017)
Fulphila	G-CSF	20 kDa	Infection during chemotherapy	2018	Aapro et al. (2017)
Asparlas	L-asparaginase	31–39 × 5 kDa	Leukemia	2018	Vrooman et al. (2021)
Esperoct	Recombinant antihemophilic factor	40 kDa	Hemophilia A	2019	Takedani and Hirose (2015)
Ziextenzo	G-CSF	20 kDa	Infection during chemotherapy	2019	Aapro et al. (2017)
Nyvepria	G-CSF	20 kDa	Neutropenia Associated with Chemotherapy	2020	Aapro et al. (2017)
Besremi	Interferon	40 kDa	Polycythemia vera	2021	Verstovsek et al. (2022)
Skytrofa	Human growth hormone	4 × 10 kDa	Growth hormone deficiency	2021	Lamb (2022)
Empaveli	Pentadecapeptide	40 kDa	Paroxysmal nocturnal hemoglobinuria (PNH)	2021	Hoy (2021)
Fylnetra	G-CSF	20 kDa PEG, N-terminal methionine	Neutropenia	2022	Yang and Kido (2011)

## 7 Conclusion

The therapeutic potential of TPPs is widely recognized; however, their short circulating half-life, poor pharmacokinetics, rapid internal clearance, and high immunogenicity present significant challenges. PEG modification has emerged as a crucial approach to address these clinical limitations of TPPs and has garnered considerable attention in the biotechnology and biomedicine field. In this review, we provide a comprehensive overview of PEG properties, modification sites, factors influencing PEGylation response, typical cases of PEG-modified TPPs, and the future prospects of PEGylated TPPs. Furthermore, we emphasize that PEGylation offers promising benefits such as prolonged half-life, enhanced tumor accumulation, improved efficacy profiles, etc. Nevertheless, there remains ample room for further exploration and innovation in this field:

i) Future research aims to further investigate the mechanism of PEGylation and its impact on the structural and functional aspects of TPPs. Researchers should strive to elucidate the underlying principles governing PEG-TPP conjugation, ensuring optimal bioactivity and stability of resulting drugs.
ii) The variability in sizes and structures of PEG poses a significant challenge. Exploring different MWs and structures of PEG can provide valuable insights into the effects of these variables on drug delivery, biodistribution, and pharmacokinetics. By investigating these factors, researchers can optimize the PEGylation process to maximize therapeutic potential while minimizing side effects.
iii) Another area of exploration should involve the development of innovative PEGylation strategies. Current methods primarily rely on the conjugation of PEG to the TPP molecule through reactive chemistry. While effective, this approach may encounter specific limitations such as restricted site specificity and potential modification of drug functionality. Therefore, researchers may focus on exploring alternative PEGylation techniques, including enzymatic or chemoenzymatic approaches, to overcome these limitations and enhance drug efficacy. Additionally, researchers are investigating the utilization of other polymer systems, such as polypropylene glycol, poly(ethylene oxide-co-propylene oxide) or carboxybetaine, as substitutes for PEG.
iv) It is crucial to comprehend the impact of PEGylation on immune response. While PEGylation of TPPs generally enhances drug circulation time and reduces immunogenicity, certain studies have reported potential immunotoxicity associated with PEG-modified drugs. Therefore, it is imperative for researchers to delve deeper into the immunological aspects, investigating the mechanisms underlying the observed immune responses and striving towards strategies that mitigate any undesirable reactions.

By focusing on these key areas, researchers can advance the field of TPPs, fostering the development of more efficient and safer PEG-modified options.
